# Clade Ib Mpox in the Democratic Republic of the Congo (DRC): Clinical and Virological Report of the First Case in Kinshasa, the Capital City

**DOI:** 10.3390/v17101327

**Published:** 2025-09-30

**Authors:** Franck Kasongo-Mulenda, Sylvie Lundi-Kizela, Sabrina Kalonji-Tshilomba, Deluxe Nsambayi-Lukusa, Mohesa Iteke, Richard Nkwembe-Mpileng, Abraham Muswibwe, Meris Matondo-Kuamfumu, Anguy Makaka, Junior Bulabula-Penge, Servet Kinbonza, Emile Malembi, Cris Kacita, Robert Shongo Lushima, Hélène Grace Otema-Akenda, Emmanuel Lokilo-Lofiko, Elisabeth Pukuta-Simbu, Adrienne Amuri-Aziza, Eddy Kinganda-Lusamaki, Prince Akil-Bandali, Ahidjo Ayouba, Martine Peeters, Eric Delaporte, Jean-Jacques Muyembe-Tamfum, Placide Mbala-Kingebeni, Antoine Nkuba-Ndaye, Véronique Kakiesse-Musumba, Steve Ahuka-Mundeke

**Affiliations:** 1 Institut National de Recherche Biomédicale (INRB), Kinshasa P.O. Box 1197, Democratic Republic of the Congo; 2 Service de Dermatologie, Département de Spécialité, Cliniques Universitaires de Kinshasa, Université de Kinshasa, Kinshasa P.O. Box 127, Democratic Republic of the Congo; 3 Service d’Ophtalmologie, Département de Spécialité, Cliniques Universitaires de Kinshasa, Université de Kinshasa, Kinshasa P.O. Box 127, Democratic Republic of the Congo; 4 Service de Microbiologie, Département de Biologie Médicale, Cliniques Universitaires de Kinshasa, Université de Kinshasa, Kinshasa P.O. Box 127, Democratic Republic of the Congo; 5 Département de Biologie Médicale, Université Protestante au Congo, Kinshasa P.O. Box 4745, Democratic Republic of the Congo; 6 Hemorrhagic Fever and Monkeypox Program, Ministry of Health, Kinshasa P.O. Box 3088, Democratic Republic of the Congo; 7 TransVIHMI (Recherches Translationnelles sur le VIH et les Maladies Infectieuses), Université de Montpellier (UM), Institut de Recherche pour le Développement (IRD), Institut National de Santé et de Recherche Médicale (INSERM), 34090 Montpellier, France

**Keywords:** follow-up, clade Ib mpox, first case, keratitis, Kinshasa

## Abstract

The ongoing mpox clade Ib outbreak was first detected in the eastern Democratic Republic of Congo (DRC) and was associated with sexual transmission. It emerged in Kamituga, a mining city and spread rapidly in surrounding health zones and reached cities like Bukavu and Goma. Here, we describe the clinical, epidemiological, and virological characteristics of the first case of clade Ib in Kinshasa, the capital city in the western DRC. The case involved a young adult woman from Kinshasa who reported unprotected sexual contact with an occasional partner, a former friend, and subsequently developed genital lesions, including vesicles and pustules. These lesions evolved and spread to the entire body, including the limbs, eyes, and soles. The diagnosis was confirmed by PCR and sequencing allowed us to assign clade Ib. We show that infection with mpox clade Ib through sexual transmission can lead to limbal nodular keratoconjunctivitis and focal conjunctivitis as complications. Importantly, these results suggest that clade Ib may have been circulating silently in Kinshasa prior to the official declaration by the Ministry of Health. This also raises concerns about the potential risk of global spread, as is currently being observed. Further studies are needed to investigate whether subsequent outbreaks of clade Ib in Kinshasa may have emerged independently of introductions from Kivu, pointing to a more complex pattern of co-circulation that could define the mpox epidemic in the capital.

## 1. Introduction

Since 2005, there have been eight Public Health Emergency of International Concern (PHEIC) declarations. The two last are related to mpox outbreaks, the first one from 2022 to 2023 with clade IIb that spread globally in more than 100 countries, and more recently since 14 August 2024 an outbreak with clade Ib [1]. The current PHEIC followed the declaration of a Public Health Emergency of Continental Security (PHECS) by Africa CDC on 13 August and both were consecutive to the concerning expansion of mpox in the DRC, especially clade Ib, firstly described in September 2023 in Kamituga, a mining city in the South Kivu province and driven by transmission through sexual contact [2,3,4]. In 2024, 14,053 confirmed mpox cases were reported in the DRC. Approximately 70% of these confirmed cases were concentrated in three provinces, including South Kivu, Equateur, and Kinshasa which accounted for 1299 cases (~10%). Kinshasa is one of the most important cities in the DRC and in Africa with a population of 15 million, according to the United Nations’ World Urbanization Prospects (2024 revision). The first cases described in Kinshasa were all clade Ia and documented since August 2023. They were linked to strains circulating in Equateur, Sud-Ubangi, and Mai-Ndombe’s provinces [5].

Here, we describe the epidemiological, clinical, and virological characteristics of the first known case of the clade Ib mpox virus (MPXV) infection reported in Kinshasa, DRC.

## 2. Materials and Methods

### 2.1. Overview of Mpox Surveillance and Data Collection in the DRC

As a reportable disease in the DRC, mpox is included in the nationwide Integrated Disease Surveillance and Response (IDSR) system. When there is an alert, an investigation is conducted to confirm that the description corresponds to the mpox case definition according to national and WHO guidelines and categorize the case as a suspected mpox case or not. Biological specimens such as skin lesion swabs (vesicle/crust) and/or other samples, are collected, sometimes with a narrative, by a multidisciplinary team using a national case investigation form (Supplementary Form S1). This form includes questions about demographic characteristics (age, sex, residence, including health zone and province, profession, and nationality), time of onset of clinical symptoms, location of lesions, type of sample, and sampling date.

### 2.2. Clinical Evaluation and Follow-Up of the Suspected Mpox Case

Clinical lesions were assessed directly through physical examination, and photographs were taken using a Nikon camera or a Samsung Z-Flip smartphone to allow better lesion description through zooming. The case was followed up over two weeks to monitor the clinical evolution and the response to the antiviral treatment. We also performed an ophthalmological examination with a magnifying lens to assess reported eye lesions and complications using a slit lamp and a blue light in the specialized unit at the Cliniques Universitaires de Kinshasa (CUK). Ophthalmologic assessment was performed under a slit lamp to emphasize ocular eruptive lesions by a fluorescein 0.5% drop test, providing a green color for the perilimbic and conjunctival lesions, with a blue (cobalt) filter. Wearing gloves, a drop of fluorescein was placed in the lower conjunctival sac. The patient blinks several times within a few seconds to distribute the dye evenly.

### 2.3. Sample Collection and Laboratory Analysis

Mucocutaneous swabs were collected from vaginal pustulobullous lesions and ophthalmic secretions. Onsite mpox diagnosis was performed using an MPXV antigen rapid test kit (RDT) (Conti Pharma^®^, Liège, Belgium), and viral DNA detection using a polymerase chain reaction (PCR) (LightMix^®^ Modular Monkeypox Virus, Roche, Basel, Switzerland) was performed at the National Institute of Biomedical Research (INRB), in Kinshasa. For sequencing, multiplex PCR amplifications were performed using MPXV clade IIb-specific primers (PrimalScheme for reference genome MT903345) [6], which generate 2.5 kb amplicons covering the complete MPXV genome. The PCR products were then quantified using a Qubit device with a Qubit^TM^ 1X dsDNA High Sensitivity Assay kit (ThermoFisher Scientific, Waltham, MA, USA). The sequencing libraries were prepared using a covidseq kit on an Illumina platform and loaded on the Miseq. The CZID consensus pipeline allowed genome consensus generation. A squirrel pipeline (Some QUIck Reconstruction to Resolve Evolutionary Links) was used for APOBEC3 analysis (https://github.com/aineniamh/squirrel, accessed on 5 June 2025). Phylogenetic analysis was conducted using a nextstrain clade I pipeline (nextstrain.org).

In addition, ophthalmic secretions were collected and shipped to the bacteriology laboratory of the CUK for bacteriological culturing using general media (blood agar, MacConkey agar, mannitol Salt agar, and thioglycolate), and the identification of the bacteria was performed using the minimal Leminor Gallery. We also performed the diffusion-based solid phase antibiotic sensitivity test using Muller–Hinton agar medium.

## 3. Case Description

### 3.1. Epidemiological Investigation and Clinical Characterization

**Figure 1:**
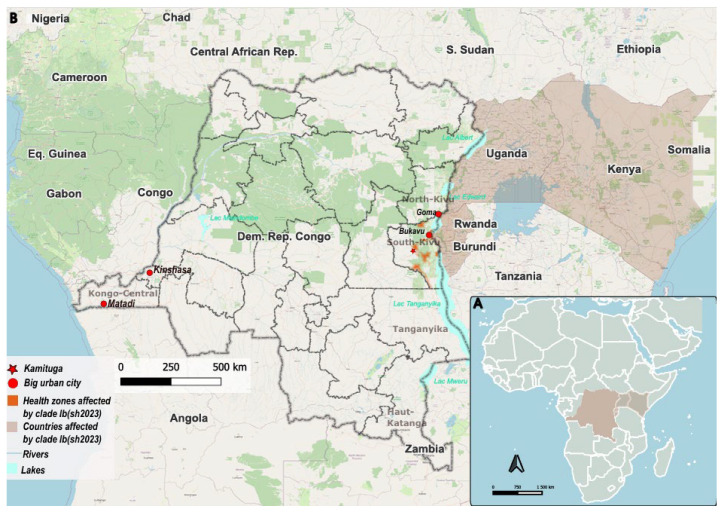
The DRC and other countries affected by clade Ib by July 2024. (**A**) Map of Africa showing the DRC and Eastern African countries where clade Ib has been identified by the investigation period (**B**) big urban cities (such as Matadi, Kinshasa, Goma, and Bukavu in red circles), Kamituga mining city (red star), and clade Ib-affected health zones.

On 1 July 2024, we investigated a woman in her twenties (case) in Kinshasa who presented mpox-like lesions, especially vesicles and pustules. The patient reported no contact with living or dead animals nor consumption of bushmeat (smoked). However, she reported occasional sexual contact with a former friend in Kinshasa prior to the start of the lesions. The casual partner, who lives in Matadi city, 360 km west of Kinshasa, in Kongo—Central province (Figure 1), reported a rash three days after the lesions appeared on the case, and he had already traveled to another African country not well-identified by the index case.

On 14 June 2024, the patient reported genital pruritus and a nodulocystic lesion in the genital area, which progressed into vesicular and pustular lesions. These initially appeared on the right labia before spreading to the limbs and other parts of the body. Symptoms included unilateral inguinal lymphadenopathy, mild vulvar discomfort, and mucosal irritation with fluid discharge. Her clinical history revealed recurrent vaginal lesions, frequent use of skin-lightening cosmetics, and a laparotomy in 2023 for an appendectomy followed by a cystotomy. Despite multiple clinic visits, her symptoms persisted. Treatment with injectable antibiotics and vaginal suppositories was unsuccessful.

A multidisciplinary team composed of dermatologists, epidemiologists, biologists, and ophthalmologists investigated the case on the eighteenth day of infection. At that moment, she presented eruptions that started in the genital area and generalized to the face and trunk.

**Figure 2:**
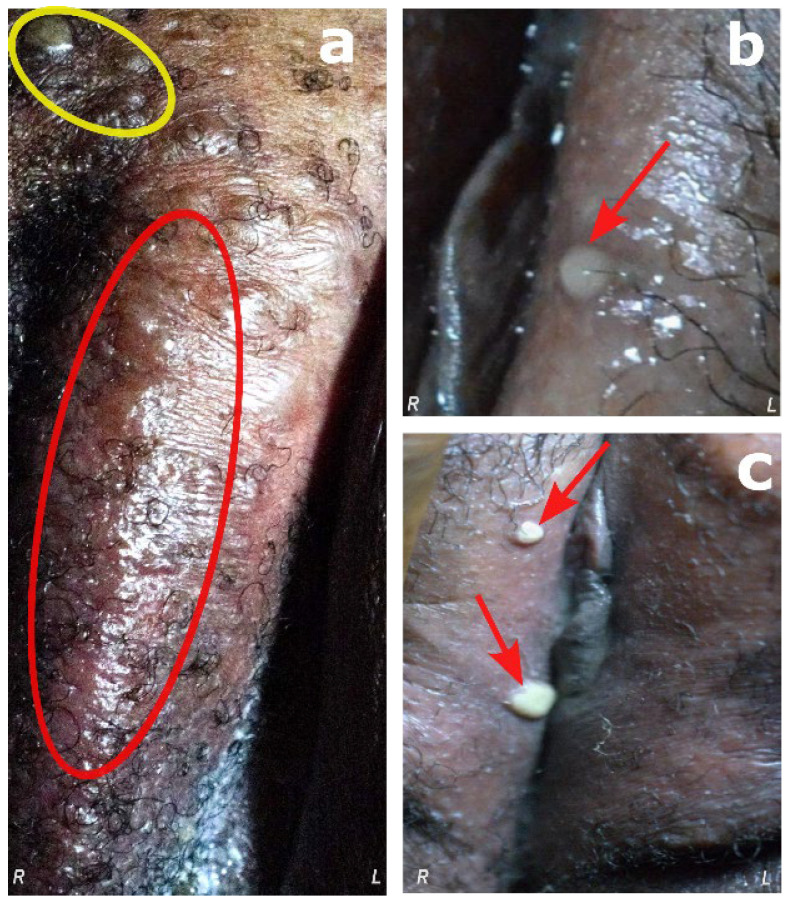
Labia majora, associated with a swelling mimicking lymphedema of the labia with few isolated opalescent bullous lesions on day 17 (1 July 2024). (**a**) Bullous lesions covered the labia majora, extending slightly to the left labia majora over the pubis (yellow circle) and an erythematous and scaly placard covered almost the entire perineo-igunino-crural region, with a whitish coating at the bottom of two inguino-crural folds (red circle). (**b**,**c**) Isolated opalescent bullous lesions (red arrows) over hung the labia majora, extending slightly towards the left labia majora. Left and right sides are indicated, respectively, by L and R.

**Figure 3:**
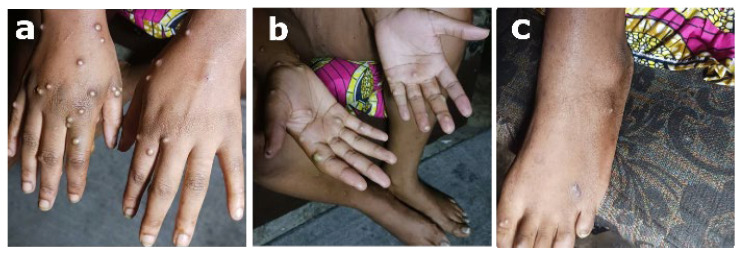
Hands, palms, and back and sole lesion monomorphism dominated by pustules. (**a**) Asymmetrical repartition of lesions on the back of hand showing multiple pustules at the same stages of evolution at the same level of progression; pustular lesions were strongly tense and surrounded by hyperpigmentation and edema for the most part. (**b**) Multiple asymmetrical pustules, with high density of lesions in relation to the spaces of the palms; strong tendency to confluence or the grouping of lesions with a few coalescing in the form of pseudobullous lesions on the palms. (**c**) Multiple pustules isolated at the same stage of evolution on the back of the feet.

There were noticeable bullous lesions in the vaginal area (Figure 2), and the lesions were asymmetrically distributed (Figure 3) and thicker on the extremities (palms and soles) than on the trunk. Significant painful edema of the labia majora and tense and occasionally confluent pustules were linked to the vaginal lesions (Figure 2).

**Figure 4:**
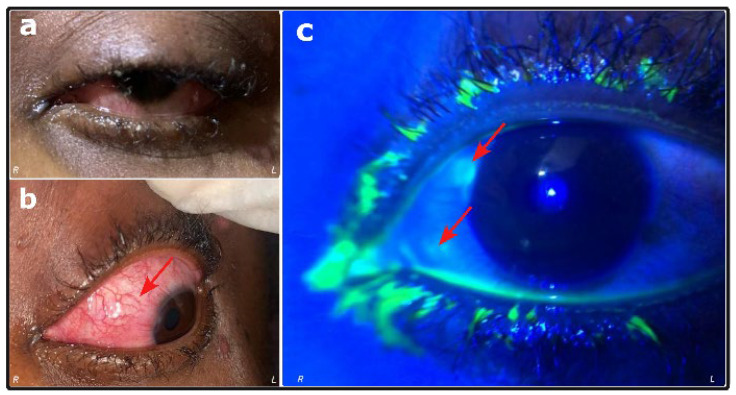
Observed lesions on the left eye (**a**) diffuse conjunctival hyperemia inferiorly, clear cornea, and abundant mucopurulent secretions in the conjunctival cul-de-sac adhering to the eyelashes (**b**) subconjunctival para-limbic nodule (red arrows) located in the supero-nasal quadrant, and two others in the supero- and infero-nasal quadrants (**c**) fluorescein test was positive on peri-limbal mucocorneal lesions and submucosal nodules (red arrows).

A rapid diagnostic test (RDT) for mpox was performed onsite and confirmed the infection. In addition, an HIV rapid diagnostic test was conducted, which yielded a negative result, indicating that the mpox case was immunocompetent. For this case, approximately 300 active lesions, vaginal edema (Figure 2), ocular disturbances (Figure 4a,b), and mobility problems caused by plantar lesions were observed on the investigation day. Discontinuities in tissue were identified with a slit lamp equipped with a cobalt blue filter; greenish lesions (very bright green) suggest the presence of epithelial lesions (conjunctival and corneal) (Figure 4c).

### 3.2. Laboratory Findings

Swab samples were taken on skin lesions, ocular secretions, vaginal pustulobullous lesions, and on hand vesicles. The mpox rapid test performed on site was positive for two samples (vesicular lesions and vaginal and hand skin swabs), and the PCR conducted later on the same samples returned with a positive result. An additional PCR conducted on conjunctival swab samples was also positive.

**Figure 5:**
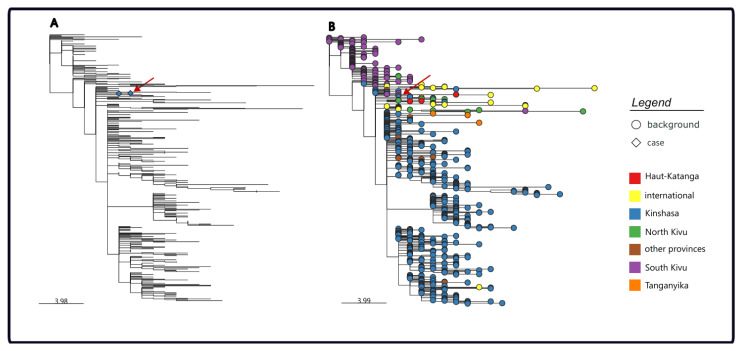
Clade I phylogenetic reconstruction using nextrain pipeline genomes showing subtree clade Ib genomes including the first Kinshasa clade Ib genomes from the case’s samples highlighted in (**panel A**) and 632 publicly available genomes included and colored by location in (**panel B**). To note, clade Ia genomes have been trimmed out. The red arrow points out the position of the genomes on the tree.

The sequences obtained from two samples (vesicle and conjunctival swabs) from the same patient were closely related, and clustered with MPXV clade Ib sequences from mpox cases detected in the South Kivu province (Figure 5). Their position in the phylogenetic tree suggests they were part of the sustained human outbreak first reported in the Kamituga health zone. The branch leading to our patient’s genomes has 10 SNPs including 6 APOBEC3-mediated mutations. However, the patient did not report traveling to the eastern part of the country or being in close contact or sexual contact with a partner coming from the eastern part of the DRC, even though this cannot completely be excluded.

Additional test results included positive cultures of ocular conjunctival secretions conducted at the University Clinics of Kinshasa’s microbiology lab, which allowed us to identify *Escherichia coli paracoli*, which was sensitive to Gentamicin, Ciprofloxacin, and Tetracycline.

### 3.3. Treatment and Management

**Figure 6:**
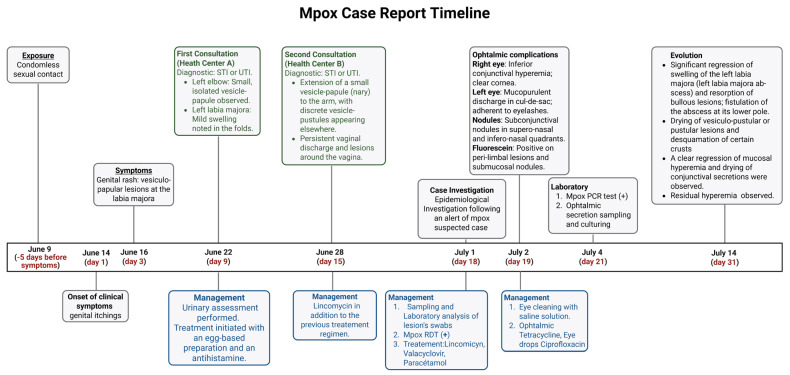
Case 1 overall timeline and main events.

Due to fear of stigmatization, the patient delayed seeking hospital care until the rash significantly worsened. In this public health investigation, our response strategy relied on three main pillars: (a) close collaboration with the national response team to implement measures aimed at preventing further transmission and to identify and monitor all high-risk contacts; (b) systematic documentation of the patient’s symptoms (fever, rash, etc.) according to the case definition in order to track their clinical progression; and (c) timely prevention and management of complications, including bacterial superinfections and ocular lesions. Standard non-pharmacological measures were implemented included maintaining good hydration and good nutritional status, making sure the patient had adequate daily protein and carbohydrate intake. Pharmacological measures consisted of standard care medication with the use of local antiseptics and dehydrating agents to reduce lesion expansion and prevent bacterial superinfection; systemic anti-inflammatory drugs to control inflammation; and analgesics used in conjunction with anti-inflammatories to manage pain. Given the high number of skin lesions, and presence of labial and ophthalmic complications in a context of lack of appropriate alternative treatment, an antiviral valaciclovir (l-valyl ester of acyclovir) was administered. The ophthalmologist’s addressed ophthalmic treatment is shown in the timeline (Figure 6).

Daily potassium permanganate baths, consisting of one 500 mg pill dissolved in 10 to 15 L of warm water (a sit bath was utilized), were also part of the treatment.

### 3.4. Overall Clinical Evolution

Evolution of mucocutaneous lesions on day 30 of the mpox infection showed a marked improvement in all lesions:

(i) Ano-genital lesions: significant regression of swelling of the left labia majora (left labia majora abscess) and resorption of bullous lesions; fistulation of the abscess at its lower pole on the tenth day of treatment with antivirals combined with antibiotics (Valaciclovir and Lincomycin); and the appearance of a more or less oval ulcerated secondary lesion.

(ii) Trunk and limb lesions: no new lesions appeared during follow-up. Existing vesiculo-pustular or pustular lesions began to dry, accompanied by the desquamation of some crusts. Additionally, certain crusted lesions evolved into flattened or depressed scar-like lesions, indicating progression toward healing.

(iii) At the ocular level, a clear regression of mucosal hyperemia and the drying of conjunctival secretions were observed. However, residual hyperemia was reported.

## 4. Discussion

This case report presents the results of an investigation on the first documented clade Ib case with in-depth clinical description and virological characterization, before the increasing number of cases that have been observed in the following weeks [7,8]. Additionally, we described the typically short-term course of clinical disease in a patient treated with antiviral medication—uncommonly used in this context—over a one-month period in the same patient who experienced complications during the acute infection. The case is the first confirmed clade Ib case in Kinshasa, the capital city of the DRC, a megalopolis of ~17 million inhabitants (https://worldpopulationreview.com/cities/dr-congo/kinshasa?utm=&utm_source=chatgpt.com (accessed on 28 May 2025)) where previous mpox confirmed cases were assigned to clade Ia [5]. Prior that period, clade Ib was strictly notified in the Kivu provinces (Nord-Kivu and Sud-Kivu) at the Eastern DRC [2,3,5,9], and in neighboring countries in the east like Burundi, Rwanda, Uganda, and Kenya. Surprisingly, during our investigation, the patient did not report a travel history to the eastern part of the country nor did they have contact with a person coming from that area. This finding suggests silent circulation of clade Ib in Kinshasa but also in the neighboring countries before July [10].

The observed concentration of lesions in the genital area for the present case, which is likely the principal entry point and associated with sexual transmission, points toward the clade Ib infection. This lesion distribution has been reported in studies showing that the mean density of skin lesions is highest in the genital area, with 89% of patients affected in this region. Unlike clade Ia, which is typically linked to zoonotic transmission and presents with lesions distributed across the body, clade Ib is commonly associated with sexual transmission, leading to a greater number of genital lesions, although severe clinical presentation is not unique to clade Ib infections [11,12]. Management strategies allowed us to reduce hesitancy and build trust with the public health team for community responses. In addition, given the severity of the disease, urgent hygiene and treatment needed to be established and seemed to be a success. Given the severity of the disease, with over 250 skin lesions observed, and the unavailability of approved antiviral agents (tecovirimat, brincidofovir, and cidofovir), the antiviral, valaciclovir, was used. Valaciclovir is well tolerated and has proven efficacy against herpes (genital) infections caused by other DNA viruses. However, current trials have reported non effectiveness of valaciclovir against MPXV [13,14], and the resolution of mpox lesions could be related to the natural progression in an immunocompetent adult. Moreover, recent findings from the PALM study on the use of tecovirimat (TPOXX) for clade I infections in mainly immunocompetent patients highlight the need to explore alternative antiviral strategies through clinical trials [15].

## 5. Conclusions

In conclusion, we presented a detailed clinical description of the first confirmed case of the MPXV clade Ib infection in Kinshasa, the capital city, in July 2024. The case was complicated by limbal nodular keratoconjunctivitis in the left eye and focal (superinfected viral) conjunctivitis in the right eye, as well as an abscess of the left labia majora in a sexually active, immunocompetent patient. Notably, the patient exhibited fragile skin in the genitocrural region, likely related to prolonged use of depigmenting topical agents. Our comprehensive clinical description underscored the distinctive features of genital involvement—both in the timing of lesion onset and the atypical morphology—as well as the risk of ocular complications. Sexual transmission appears to be the most probable route, and public health strategies should reflect this possibility. These findings suggest that MPXV clade Ib may have been circulating silently in Kinshasa prior to its official recognition by the Ministry of Health. Further studies are needed to determine whether subsequent outbreaks of clade Ib in Kinshasa occurred independently of introductions from Kivu, which would point to a more complex pattern of co-circulation shaping the mpox epidemic in the capital.

## Data Availability

The sequences obtained from both vesicle and conjunctival swabs have been made publicly available to NCBI under accession ID PQ352022 and PQ352023, respectively.

## Supplementary Materials

The following supporting information can be downloaded at: https://www.mdpi.com/article/10.3390/v17101327/s1, Form S1: case investigation form.

## Abbreviations

Africa CDC	Africa Center for Disease Control
CUK	Cliniques Universitaires de Kinshasa
DRC	Democratic Republic of the Congo
dsDNA	Double Strain Deoxyribonucleic acid
IDSR	Integrated Disease Surveillance and Response
INRB	Institut National de Recherche Biomédicale
IRD	Institut de Recherche pour le Développement
MPXV	Monkeypox Virus
PCR	Polymerase Chain Reaction
PHECS	Public Health Emergency of Continental Security
PHEIC	Public Health Emergency of International Concern
RDT	Rapid Diagnostic Test
WHO	World Health Organization
