# Supplementary material for: Clade Ib Mpox in the Democratic Republic of the Congo (DRC): Clinical and Virological Report of the First Case in Kinshasa, the Capital City
Source: Viruses. 2025 Sep 30;17(10):1327. doi: 10.3390/v17101327 (PMC12568009; doi:10.3390/v17101327)
Supplement: Supplementary file 1 [file viruses-17-01327-s001.zip › viruses-3839878-supplementary.pdf]

**REPUBLIQUE DEMOCRATIQUE DU CONGO**
**MINISTERE DE LA SANTE PUBLIQUE, HYGIENE ET PREVENTION**

**PROGRAMME NATIONAL DE LUTTE CONTRE LE MONKEYPOX ET FIEVRES HEMORRAGIQUES VIRALES**

# FICHE D'INVESTIGATION DE CAS DE MONKEYPOX

**Identifiant unique :** **Date de notification (J, M, A)** __/__/__

## 1. Statut du patient

☐ Vivant ☐ Décédé Si décédé, date du décès (J, M, A) ___/___/___

Lieu du décès (Nom du village) ________________________
Pays______________________________________________ Province : ___________________________________

Présence d'une cicatrice du vaccin contre la variole ? ☐ Oui ☐ Non ☐ Ne sais pas

## 2. Information sur le patient

Nom_____________________ Post-nom : ____________________ Prénom____________________
Date de naissance (J, M, A) ___/___/___ Âge (année, mois, jours) ______________ Sexe ☐ M ☐ F
Nationalité_______________________ Ethnicité/tribu ______________________

**Lieu de résidence permanente**
Nom du chef de la famille _______________________________
Village /Ville_________________________ Quartier (facultatif) ___________________________________
Zone de Sante _____________________________ Aire de Santé _________________________________________
Pays de résidence ________________________________________

**Profession du patient (cocher toutes les cases correspondantes)**

☐ Enfant (non étudiant)
☐ Étudiant
☐ Marchand de gibier
☐ Marchand (autre)
☐ Chasseur
☐ Médecin traditionnel
☐ Employé de maison
☐ Planteur/Fermier
☐ Mineur
☐ Personnel de santé (préciser ci-dessous) ______________
☐ Autre profession ___________________________________

**Endroit où le patient est tombé malade :**
Province _____________________Village/ville _________________________Zone de santé_____________________
Aire de santé __________________
Coordonnées GPS de la maison : Latitude_______________________ ; Longitude_____________________________

Si ce n'est pas la résidence permanente, Dates de résidence dans cet endroit : __/__/___ au __ /__/__

## 3. Symptômes et signes cliniques présentés

**Le patient a une maladie à éruption cutanée?** ☐ Oui ☐ Non OU
Date de l'apparition de l'éruption cutanée (J, M, A) : __/___/___

**Le patient a-t-il eu de la fièvre?** ☐ Oui ☐ Non ☐ Ne sais pas
Si oui, date de début de la fièvre (J, M, A) : ___/___/___
La fièvre a-t-elle précédé l'éruption cutanée? ☐ Oui ☐ Non ☐ Ne sais pas

**Si la maladie est active, avec éruption cutanée**, ou présence de cicatrices, compter le nombre de lésions ou de cicatrices situées sur les endroits suivants :

Visage : ____ Paume des mains : ____
Thorax : ____ Plante des pieds : _____
Extrémités supérieures : ____ Muqueuse génitale : ____

**Si la maladie est active,**

a. Les lésions sont-elles au même stade de développement sur tout le corps? ☐ Oui ☐ Non ☐ Ne sais pas

b. Toutes les lésions sont-elles une taille et une forme semblables? ☐ Oui ☐ Non ☐ Ne sais pas

c. Les lésions sont-elles dures et profondes? ☐ Oui ☐ Non ☐ Ne sais pas

**Faites le ressembler de lésions à:**

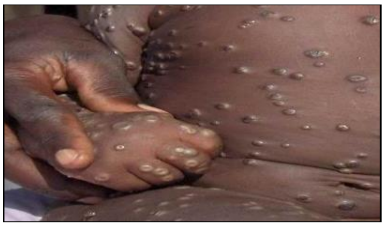

a. ☐ Oui ☐ Non

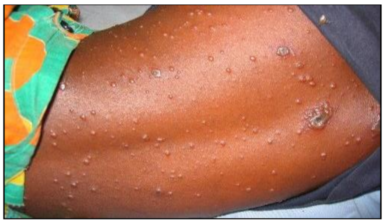

b. ☐ Oui ☐ Non

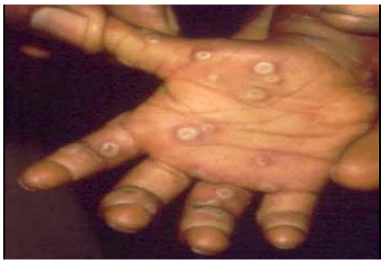

c. ☐ Oui ☐ Non

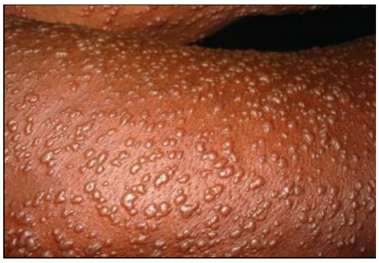

d. ☐ Oui ☐ Non

**Le patient présente-t-il ou a-t-il présenté les symptômes suivants (cocher toutes les cases correspondantes)**

| | | | |
|---|---|---|---|
| Vomissements/nausées | ☐ Oui | ☐ Non | ☐ Ne sais pas |
| Toux | ☐ Oui | ☐ Non | ☐ Ne sais pas |
| Adénopathie inguinale | ☐ Oui | ☐ Non | ☐ Ne sais pas |
| Adénopathie axillaire | ☐ Oui | ☐ Non | ☐ Ne sais pas |
| Adénopathie cervicale/sous-maxillaire | ☐ Oui | ☐ Non | ☐ Ne sais pas |
| Frissons ou sueur | ☐ Oui | ☐ Non | ☐ Ne sais pas |
| Douleur à la gorge en avalant | ☐ Oui | ☐ Non | ☐ Ne sais pas |
| Ulcères buccaux | ☐ Oui | ☐ Non | ☐ Ne sais pas |
| Maux de tête | ☐ Oui | ☐ Non | ☐ Ne sais pas |
| Lésions accompagnées de démangeaisons | ☐ Oui | ☐ Non | ☐ Ne sais pas |
| Douleur musculaire (myalgie) | ☐ Oui | ☐ Non | ☐ Ne sais pas |
| Fatigue | ☐ Oui | ☐ Non | ☐ Ne sais pas |
| Conjonctivite | ☐ Oui | ☐ Non | ☐ Ne sais pas |
| Sensibilité à la lumière | ☐ Oui | ☐ Non | ☐ Ne sais pas |
| Le patient était-il alité ? | ☐ Oui | ☐ Non | ☐ Ne sais pas |

## 4. Informations sur l'hospitalisation et complications éventuelles

Au moment de cette investigation, le malade est-il hospitalisé ? ☐ Oui ☐ Non

Si oui, Date d'hospitalisation (J, M, A) __ /__/__ Nom de l'hôpital_______________ Village/Ville __________________

Zone de Santé ________________

Le malade est-il en isolement ☐ Oui ☐ Non Si oui, Date d'isolement (J, M, A) __/__/__

Le malade était-il hospitalisé ailleurs ou a visité un centre de soins (moderne ou traditionnel) pour la maladie actuelle ?

☐ Oui ☐ Non ☐ Ne sais pas

Si oui, Veuillez compléter une ligne ci-dessous pour chacune des hospitalisations précédentes :

| Dates d'hospitalisation | Nom du centre de soins | Village/ville | Aire de Santé/Zone de santé |
|---|---|---|---|
| __/__/__ - __/__/__ (J, M, A) | | | |
| __/__/__ - __/__/__ (J, M, A) | | | |

**Le patient présente-t-il de complications ?** ☐ Oui ☐ Non

Si oui, laquelle (Cocher tout ce qui s'y réfère)

- ☐ Surinfection cutanée,
- ☐ Infection respiratoire,
- ☐ Détresse respiratoire aigüe
- ☐ Cécité
- ☐ Coma
- ☐ Insuffisance rénale
- ☐ Dénutrition/déshydratation

## 5. Facteurs d'exposition

**Le patient a-t-il été en contact avec une ou des personnes présentant des symptômes similaires durant les trois semaines précédant l'apparition des symptômes ?**

☐ Oui ☐ Non ☐ Ne sais pas

Si oui, répondez aux questions suivantes concernant ces personnes malades (indiquer toutes les personnes malades). Au besoin, utilisez le verso de la page comme espace additionnel.

Nom ________________________ Prénom ________________ Liens avec le patient_______________________

Première date de contact avec la personne malade (J, M, A) ___/___/___ Statut : ☐ Vivant ☐ Décédé

**Interaction avec cette personne (cocher toutes les cases correspondantes) :**

- ☐ Contact familial/vivent ensemble
- ☐ Ont préparé de la nourriture ensemble
- ☐ Ont partagé un lit/dormi
- ☐ S'est occupé de cette personne malade
- ☐ Camarade d'école ou de jeu
- ☐ Se sont rencontrés au marché
- ☐ Ont chassé ensemble
- ☐ Ami/relation sociale
- ☐ Ont été à l'église, ont assisté à un service religieux ensemble
- ☐ Autre_________________________________________

**Type de contact (cocher toutes les cases correspondantes)**

- ☐ Type 1 : Contact direct avec les lésions cutanées (Incluant le contact sexuel)
- ☐ Type 2 : Contact avec des secrétions/excrétions du malade (sang, vomissures, salives, urines, selles)
- ☐ Type 3 : A dormi ou mangé avec, ou séjourné dans la même maison ou pièces que le malade

**Le patient a-t-il touché un animal domestique ou sauvage ou mordu par celui-ci durant les trois semaines précédant l'apparition des symptômes?**

☐ Oui ☐ Non ☐ Ne sais pas

Si oui, quelle sorte d'animal____________________________

Date de contact ___/___/___

Type de contact (cocher toutes les cases correspondantes)

- ☐ Rongeurs vivant dans la maison
- ☐ Animal vivant provenant de la forêt
- ☐ Animal mort provenant de la forêt
- ☐ Animal acheté pour sa viande

**Le patient a-t-il voyagé en dehors de chez lui ou de son village/ville avant la maladie actuelle ?**

☐ Oui ☐ Non ☐ Ne sais pas

Si oui, Village____________ Zone de Santé____________ Date (J, M, A) __/__/__

**Le patient a-t-il été hospitalisé, a-t-il consulté dans un hôpital ou visité quelqu'un hospitalisé avant la maladie actuelle ?**

Si oui, Nom du patient__________________________ Date(s) (J, M, A) : __/__/__ au __ /__/__

Nom du centre Médical ______________________Village _________________ Zone de Santé___________________

## 6. Prélèvements d’échantillon de laboratoire réalisés

A accepté le prélèvement d’échantillon ? ☐ Oui ☐ Non Si oui, date (J, M, A) ___/___/___

Type : ☐ Croûtes ☐ Ecouvillon (swab) ☐ Prélèvement sanguin

Prélevez au moins deux échantillons de croutes ou swab provenant de deux parties différentes du corps du patient.

Tous commentaires additionnels au sujet du cas:

______________________________________________________________________________________

______________________________________________________________________________________

______________________________________________________________________________________

______________________________________________________________________________________

## 7. Classification du cas

☐ Cas suspect ☐ Cas probable ☐ Cas confirmés ☐ Non cas ☐ Cas non actif

## 8. Information sur la personne remplissant la fiche

Noms et prénoms (Lister si équipe) : ____________________________________________________________

Fonctions et institution : _________________________________________________________________

Téléphone : ________________________________Email :_______________________________________

Date de remplissage de la fiche (J,M,A) : ___ /___/___ Lieu d’investigation :_________________________________

## 9. Information sur la personne fournissant les informations (autre que le patient, si applicable)

Nom : __________________ Prénom : _____________________________ Sexe M F Age : ______________

Adresse : _______________________ Aire de Santé : ______________________ Zone de Santé :________________

Province : ________________________________ Pays : ________________________ Téléphone : ______________

Lien avec le patient : ______________________________

____________________________________________________________

Cas signalé par (cocher toutes les cases correspondantes et préciser)

- ☐ Équipe mobile, n° _________________________
- ☐ Centre de santé ___________________________
- ☐ Hôpital___________________________________
- ☐ Autre : ___________________________________
